# SIAH2 antagonizes TYK2-STAT3 signaling in lung carcinoma cells

**DOI:** 10.18632/oncotarget.1899

**Published:** 2014-04-12

**Authors:** Sylvia Müller, Yuan Chen, Torsten Ginter, Claudia Schäfer, Marc Buchwald, Lienhard M. Schmitz, Jana Klitzsch, Alexander Schütz, Andrea Haitel, Katharina Schmid, Richard Moriggl, Lukas Kenner, Karlheinz Friedrich, Claude Haan, Iver Petersen, Thorsten Heinzel, Oliver H. Krämer

**Affiliations:** ^1^ Center for Molecular Biomedicine, University of Jena, Department of Biochemistry, Jena, Germany; ^2^ Institute of Pathology, University Clinic Jena, Germany; ^3^ Department of Biochemistry, Faculty of Medicine, Giessen, Germany; ^4^ Institute of Pathology, University of Leipzig, Germany; ^5^ Clinical Institute of Pathology, Medical University of Vienna, Austria; ^6^ Institute of Anatomy and Experimental Morphology, University of Hamburg-Eppendorf, Germany; ^7^ Ludwig Boltzmann Institute for Cancer Research, Vienna, Austria; ^8^ University of Veterinary Medicine, Vienna and Medical University of Vienna, Austria; ^9^ Institute of Biochemistry II, University Clinic, Jena, Germany; ^10^ Signal Transduction Laboratory/Life Sciences Research Unit, University of Luxembourg, Luxembourg; ^11^ Department of Toxicology, University Medical Center, Mainz, Germany

**Keywords:** lung cancer, SIAH2, STAT3, TYK2, UBCH8

## Abstract

The Janus tyrosine kinases JAK1-3 and tyrosine kinase-2 (TYK2) are frequently hyperactivated in tumors. In lung cancers JAK1 and JAK2 induce oncogenic signaling through STAT3. A putative role of TYK2 in these tumors has not been reported. Here, we show a previously not recognized TYK2-STAT3 signaling node in lung cancer cells. We reveal that the E3 ubiquitin ligase seven-in-absentia-2 (SIAH2) accelerates the proteasomal degradation of TYK2. This mechanism consequently suppresses the activation of STAT3. In agreement with these data the analysis of primary non-small-cell lung cancer (NSCLC) samples from three patient cohorts revealed that compared to lung adenocarcinoma (ADC), lung squamous cell carcinoma (SCC) show significantly higher levels of SIAH2 and reduced STAT3 phosphorylation levels. Thus, SIAH2 is a novel molecular marker for SCC. We further demonstrate that an activation of the oncologically relevant transcription factor p53 in lung cancer cells induces SIAH2, depletes TYK2, and abrogates the tyrosine phosphorylation of STAT1 and STAT3. This mechanism appears to be different from the inhibition of phosphorylated JAKs through the suppressor of cytokine signaling (SOCS) proteins. Our study may help to identify molecular mechanisms affecting lung carcinogenesis and potential therapeutic targets.

## INTRODUCTION

Binding of cytokines to their cognate receptors activates receptor-associated JAKs which catalyze phosphorylation of STATs [1]. The JAK family comprises the kinases JAK1,-2,-3 and TYK2. TYK2 is the founding member of the JAK family [2]. Phosphorylated STATs enter the nucleus as homo-/heterodimers and control genes determining metazoan development and homeostasis [1].

Molecular mechanisms restricting STAT signaling rely on phosphatases and ubiquitin-dependent proteasomal degradation [3]. An E1 enzyme transfers ubiquitin molecules to E2 ubiquitin conjugases which interact with E3 ubiquitin ligases conferring substrate specificity [4, 5]. Proteins belonging to the SOCS family are induced by activated STATs to establish a negative feed-back on STAT signaling [6]. Binding studies suggest that SOCS1, SOCS3, Elongin-B/C, Cullin-2/5, and RBX build ubiquitin ligase complexes that enhance the proteasomal degradation of phosphorylated JAK2 [7, 8]. Particularly disease-associated, constitutively phosphorylated mutants of JAK2 are prone to SOCS-dependent proteasomal elimination [7]. Nonetheless, interaction of SOCS1 with Elongins is dispensable for SOCS-dependent inhibition of JAK2 and overexpressed Elongins/SOCS1 do not destabilize wild-type JAK2 in cancer cells [9]. There are also very modest differences in JAK2 expression between wild-type and SOCS1 null mice [7-9]. Via its SH2 domain SOCS1 can interact with TYK2 phosphorylated at phospho-tyrosines 1054 and 1055. This mechanism also determines IFNα-receptor (IFNAR) surface expression [10]. It has not been defined which ubiquitin conjugase(s) and -ligase(s) catalyze the turnover of unphosphorylated TYK2 in resting cells.

Tumors often have constitutively active STAT signaling [11, 12]. This also holds true for lung cancer, which is the leading cause of cancer-related deaths worldwide [13, 14]. Therefore, it is important to delineate the biological roles of JAK-STAT signaling in healthy and diseased lungs. Whereas it was found STAT3 acted as a JAK1/JAK2-induced oncogenic driver in lung cancers [15-18], STAT1 expression was reported to form part of a genetic signature predicting event-free and overall survival of lung cancer patients [19].

SIAH proteins (human homolog of *Drosophila* Seven-In-Absentia) are efficient ubiquitin ligases. Their contribution to cell fate is discussed controversially and might be cell type-dependent [5, 20]. Limited information is available on the roles of TYK2 and SIAHs in diseased lungs. Here, we reveal that TYK2 induces STAT3 activation and that TYK2 is a SIAH2 target. Increasing SIAH2 levels by overexpression and by activation of p53, as well as the induction of its associated E2 ubiquitin conjugase UBCH8 by interferon-α (IFNα), is linked to degradation of TYK2. Moreover, we demonstrate a significant association of SIAH2 expression with lung SCC. SIAH2 levels inversely correlate with STAT3 phosphorylation and metastatic gene expression in NSCLC.

## RESULTS

### SIAH2 promotes proteasomal degradation of TYK2

**Fig 1:**
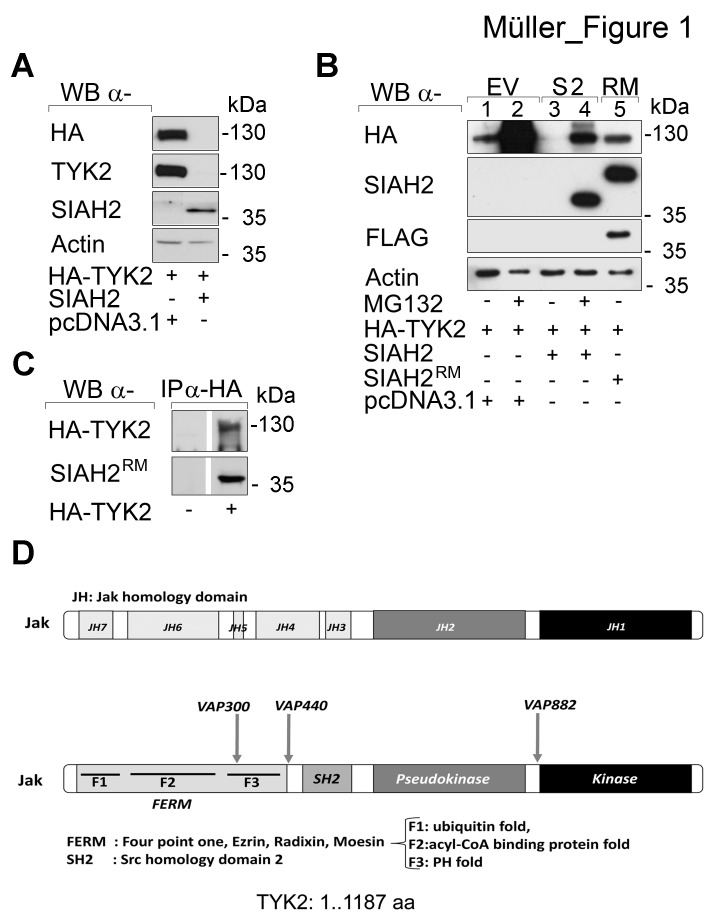
Catalytically intact SIAH2 reduces TYK2 protein levels (A) HEK293T cells were transfected with vectors encoding HA-TYK2 (1 µg), wild-type SIAH2 (0.5 µg) or empty vector pcDNA3.1 (0.5 µg) instead of SIAH2. Cell lysates were analyzed by Western blot (WB; α, anti) as indicated; kDa corresponds to Mr according to protein ladder standards (Fermentas SM1811). (B) HEK293T cells were transfected with HA-TYK2, SIAH2, or mutant FLAG-RM-SIAH2^H99A/C102A^ (SIAH2^RM^) [58]. Empty vector pcDNA3.1 was used to keep the amount of transfected DNA constant. After 24 h cells were treated with MG132 (+, 16 h, 2 µM) to preserve protein ubiquitinylation. Western blot analyses were done as indicated. (C) HEK293T cells were transfected with HA-TYK2 and mutant FLAG-RM-SIAH2^H99A/C102A^ (SIAH2^RM^) [58]. Inactive SIAH2 had to be used due to the potent proteasomal elimination of TYK2 by wild-type SIAHs. HA-TYK2 was immunoprecipitated (IP) with α-HA antibody (-, IP from untransfected cells). Co-precipitated mutant SIAH2 and TYK2 were detected by immunoblot. (D) SIAH binding motifs (VxP) are contained within the TYK2 protein sequence.

Previously, we reported that the E3 ubiquitin ligase SIAH2 promotes the proteasomal degradation of the mutant receptor tyrosine kinase (TK) FLT3-ITD in leukemic cells and of the non-receptor TK ACK1 in breast cancer cells [21, 22]. When we tested the impact of SIAH2 on the TK TYK2, we found that ectopic expression of SIAH2 in human embryonic kidney cells (293T cells) and in human lung adenocarcinoma H1299 cells strongly decreased the levels of TYK2 (Fig. 1A and 3B). These findings argue for a SIAH2-induced degradation of TYK2 *in vivo*. Remarkably, the effect of SIAH2 on TYK2 was specific, as there was no loss of JAK1 in H1299 and 293T cells with increased expression of SIAH2 (Supplemental Fig. S1).

SIAH2 is an E3 ubiquitin ligase catalyzing proteasomal degradation of most of its substrates [5, 20]. Therefore, we tested with a chemical inhibitor and with a SIAH2 loss-of-function approach whether SIAH2 promotes proteasomal degradation of TYK2. The proteasomal inhibitor MG132 stabilized TYK2 dramatically, which demonstrated its high basal turnover (Fig. 1B, lanes 1 vs. 2). Moreover, treatment with MG132 preserved TYK2 when SIAH2 was co-expressed (Fig. 1B, lanes 3 vs. 4). To ensure that the catalytic activity of SIAH2 accelerates the turnover of TYK2, we overexpressed SIAH2 or an inactive RING mutant of SIAH2 with TYK2. Indeed, degradation of TYK2 required the intact E3 ubiquitin ligase activity of SIAH2 (Fig. 1B, lanes 1, 3, 5).

Having assessed that SIAH2 induces proteasomal degradation of TYK2, we tested whether SIAH2 is detectable in TYK2 immunoprecipitates (IPs). We could readily detect SIAH2 in anti-TYK2 IPs (Fig. 1C). Inspection of the TYK2 protein discloses several VxP SIAH consensus binding motifs (Fig. 1D), which allow recognition by SIAHs and ubiquitin-dependent proteasomal degradation [5, 20].

### Interaction between TYK2 and UBCH8

The E2 ubiquitin conjugase UBCH8 interacts with SIAHs [22, 23]. The physiological stimulus IFNα induces a STAT1-dependent transcription of the *UBE2L6* gene and a subsequent accumulation of UBCH8 in cells [24, 25]. Therefore, we assessed UBCH8's putative role in the proteasomal degradation of TYK2.

**Fig 2:**
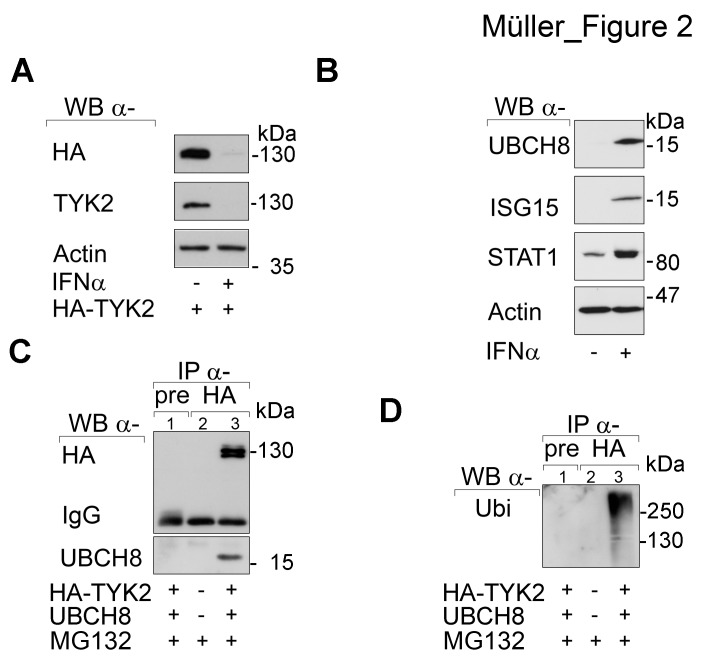
SIAH2 interacts with UBCH8 (A) HEK293T cells were transfected with HA-TYK2 and after 24 h the cells were treated with 10^3^ U IFNα for another 24 h. Western blot was done as indicated. (B) HEK293T cells were treated with 10^3^ U IFNα for 24 h. Western blot was done as stated. (C and D) HEK293T cells were transfected with HA-TYK2 and UBCH8-V5 (+/+). After 24 h cells were treated with MG132 (+, 16 h 10 µM). HA-TYK2 was immunoprecipitated with α-HA antibody (lane 3). Membranes were probed for HA and V5. The upper part of the membrane was then reprobed for ubiquitin (right panel). Pre-immune serum IP formed with an equivalent amount of lysate from transfected 293T cells (lane 1; pre) and HA-IP with untransfected HEK293T cell lysates (lane 2) served as negative controls.

When we induced UBCH8 with IFNα we found that prolonged stimulation reduced endogenous and overexpressed TYK2 (Fig. 2A). We could verify the induction of UBCH8 and of other IFN/STAT1 targets (ISG15 and STAT1 itself) in the IFNα-treated cells (Fig. 2B).

Next, we tested whether UBCH8 occurs in IPs formed with an antibody directed against TYK2. Indeed, UBCH8 was present in anti-TYK2 IP complexes (Fig. 2C). Moreover, TYK2 was strongly ubiquitinylated in such IPs (Fig. 2D).

As poly-ubiquitinylation marks proteins for proteasomal degradation, these data are consistent with the rapid proteasomal degradation of TYK2 (Fig. 1B). The increased expression of UBCH8 in response to IFNs and the proteasomal degradation of TYK2 may create a negative feed-back loop on STAT signaling.

### SIAH2 inhibits a TYK2-STAT3 signaling hub

Lung cancers often carry constitutively active tyrosine phosphorylated STAT3 (abbreviated as pSTAT3) induced by JAK1 or JAK2 and the JAK2-STAT3 signaling node is a major oncogenic driver in lung tumors [15-18, 26]. We asked whether TYK2 evokes STAT3 signaling in lung cancer cells and if SIAH2 can attenuate this process.

**Fig 3:**
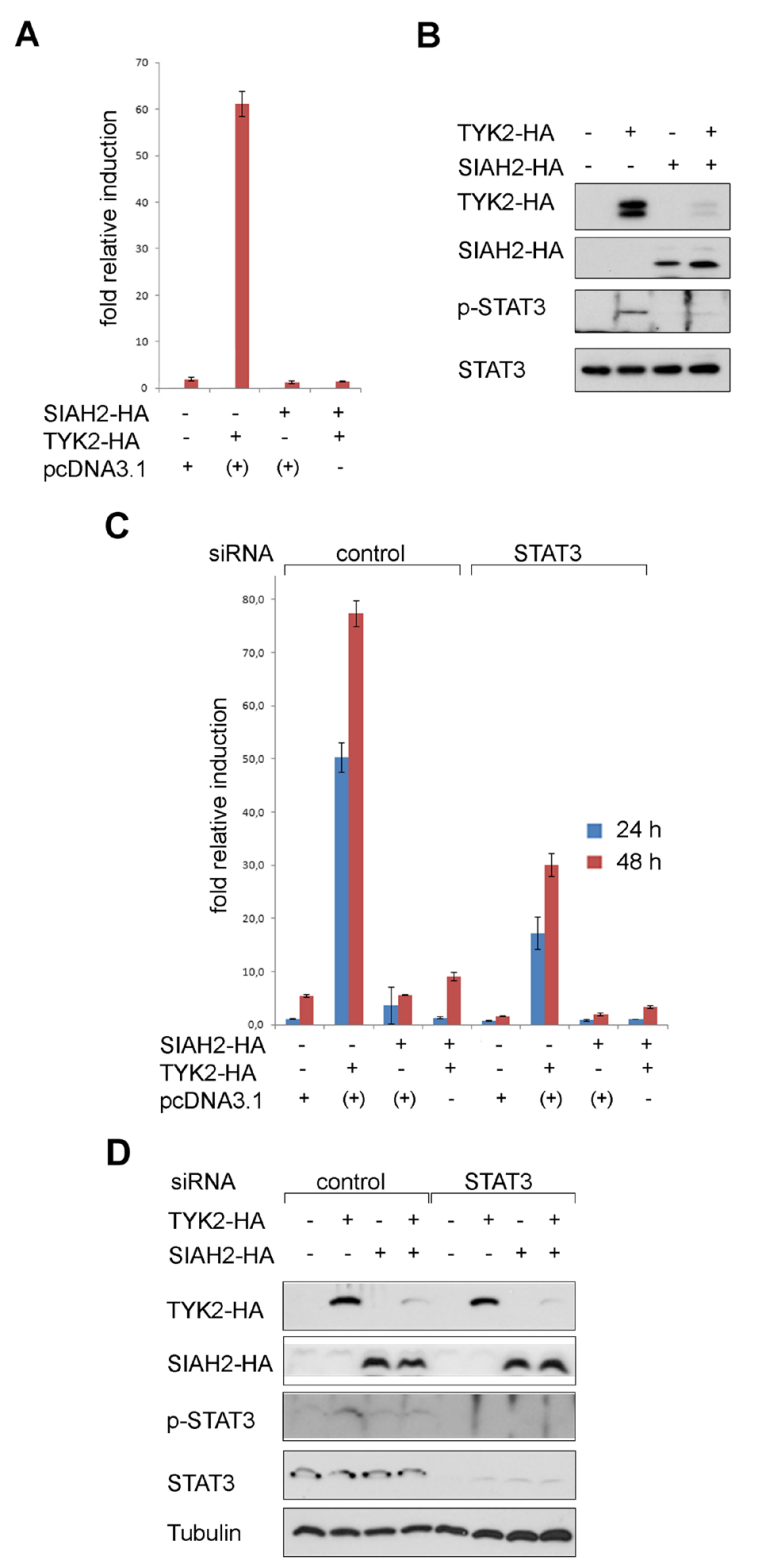
SIAH2 blocks TYK2 signaling to STAT3 in lung cancer cells (A) H1299 cells were transfected with a GAS-Luc reporter construct, HA-SIAH2, and HA-TYK2; +: pcDNA3.1 was used instead of SIAH2 or TYK2 to keep the amounts of DNA constant. Luciferase activity measured 48 h after transfection was normalized to β-Gal activity (fold relative induction). (B) The extracts used for luciferase assays (A) were probed for HA-tagged SIAH2 and TYK2, tyrosine phosphorylated STAT3 and total STAT3 protein levels by Western blot. (C) H1299 cells were transfected with a GAS-Luc reporter construct, HA-SIAH2, and HA-TYK2; pcDNA3.1 was used to keep the amounts of DNA transfected constant. Luciferase activity measured 24 h and 48 h after transfection was normalized to β-Gal activity. STAT3 was eliminated by siRNA.(D) The extracts used for luciferase assays (C) were probed for HA-tagged SIAH2 and TYK2, tyrosine phosphorylated STAT3 and total STAT3 protein levels by Western blot; tubulin was used as a loading control.

To answer this question we investigated whether the SIAH2-induced degradation of TYK2 affects transcriptional activation of a luciferase reporter system containing binding sites for STAT1/STAT3 homo- or heterodimers (GAS-Luc) in H1299 cells. Overexpression of TYK2 induced this reporter encoding luciferase and concomitant expression of SIAH2 strongly suppressed reporter activation (Fig. 3A). This interaction between TYK2 and SIAH2 could also be seen with a STAT1/STAT2-dependent ISRE-Luc reporter (Fig. S2).

The expression of TYK2 became reduced when SIAH2 was co-transfected into H1299 cells (Fig. 3B). Together with the elimination of TYK2, the induction of pSTAT3 upon expression of TYK2 disappeared. These data suggest that SIAH2 dampens the TYK2-induced phosphorylation of STAT3 through catalyzing the proteasomal degradation of TYK2. The levels of STAT3 and STAT1 though remained stable (Fig. 3B and data not shown).

The ambivalent role of STAT1 and STAT3 in lung cancer [15-19] prompted us to analyze if the TYK2-dependent activation of the reporter is mediated by STAT1 or STAT3. We knocked-down STAT3 with a very efficient siRNA in H1299 cells and found that the induction of GAS-dependent transcription relied on the presence of STAT3 (Fig. 3C and 3D).

By Western blotting we confirmed that increasing SIAH2 levels diminished TYK2 expression (Fig. 3D). We also overexpressed TYK2 and SIAH2 in U3A fibrosarcoma cells which are devoid of STAT1. STAT3 was also sufficient for the activation of the GAS-Luc reporter by TYK2 in these cells (data not shown).

To this end we demonstrate a novel link between TYK2 and STAT3. Moreover, we show that SIAH2 reduces TYK2 and the TYK2-dependent activation of STAT3 in lung cancer cells.

### Induction of the tumor suppressor p53 activates SIAH2 and reduces TYK2

The tumor suppressive transcription factor p53 has been reported to induce SIAH1 in various cell types [27]. In breast cancers, increased SIAH2 expression is associated with p53 [28].

**Fig 4:**
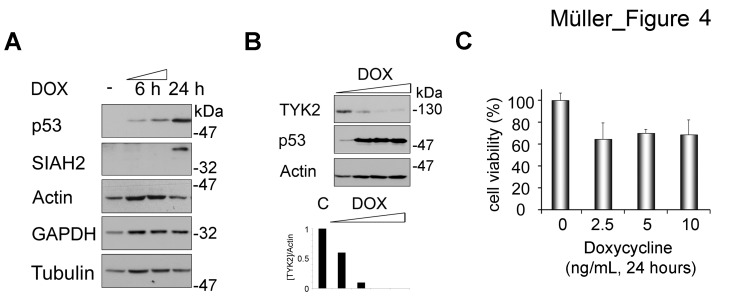
Induction of SIAH2 by p53 is linked to a decrease of endogenous TYK2 (A) H1299 cells were treated with doxycycline (DOX, 5 ng/ml; -, untreated cells) for 24 h. Induction of p53 and of SIAH2 were analyzed by Western blot. Actin, tubulin, and GAPDH serve as independent loading controls. (B) H1299 cells were treated with increasing amounts of doxycycline (DOX, 2.5/5/10/25 ng/ml; C, untreated cells) for 24 h. Induction of p53 and SIAH2 degradation of TYK2 were analyzed by Western blot (WB). Diagram below blots shows densities for [TYK2] divided by [Actin] (loading control). (C) MTT assay was used to evaluate the effect of doxycycline treatment on cytotoxicity in H1299 cells. Cells were stimulated as indicated and cell viability was measured as conversion of MTT by intact H1299 cells (untreated control cells were set as 100%).

To test whether p53 causes an upregulation of SIAH2 in lung cancer cells, we used H1299 cells stably carrying a p53 transgene that can be induced with doxycycline [29]. We noted an accumulation of SIAH2 in H1299 lung adenocarcinoma cells with doxycycline-inducible p53 (Fig. 4A). This finding extends previous observations on a p53-dependent induction of SIAH1 [27].

Moreover, we noted that p53 activation inducing SIAH2 decreased TYK2 levels (Fig. 4B). This attenuation of TYK2 occurred before and not as a mere consequence of p53-dependent apoptosis (Figs. 4C and S3).

These results illustrate that an increased expression of p53 and SIAH2 is associated with decreased levels of TYK2 in lung cancer cells.

### Induction of the tumor suppressor p53 suppresses basal and IFNα-induced STAT signaling

**Fig 5:**
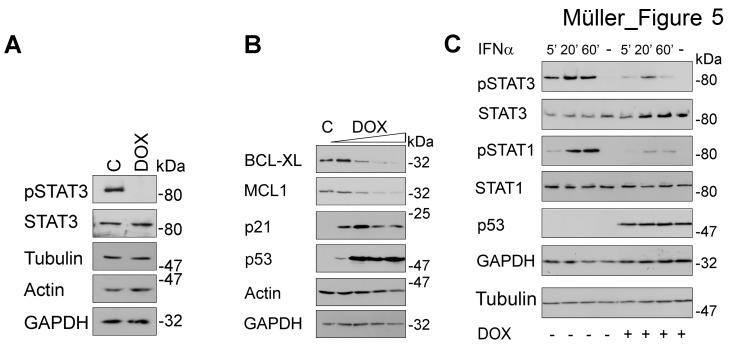
Induction of p53 blocks STAT signaling (A) H1299 cells were incubated with doxycycline (DOX, 5 ng/ml; C, untreated cells) for 24 h. Phosphorylated and total STAT3 levels as well as three loading controls were assessed by immunoblot. (B) H1299 cells were treated as described in Figure 4B. Western blot analyses evaluated the levels of the STAT3 target genes BCL-XL and MCL1 as well as of p53 and its target gene p21; actin and GAPDH were used as independent loading controls. (C) H1299 cells were treated with DOX (+, 10 ng/ml, 24 h) or left untreated (-). Subsequently, cells were stimulated with IFNα (+, 10^3^ U/ml; -, unstimulated) for the times indicated. Cell extracts were analyzed by immunoblot as indicated (kDa, Mr according to protein ladder standards: NEB P7708 or Fermentas SM1811).

H1299 cells harbor low levels of constitutively active pSTAT3 [30], which allowed analyzing whether p53-mediated degradation of TYK2 represses basal STAT3 phosphorylation. Induction of p53 readily abolished phosphorylation of STAT3, while total STAT3 levels remained unaffected (Fig. 5A).

Since pSTAT3 induces anti-apoptotic genes involved in tumorigenesis and chemotherapy resistance [15], inhibition of its phosphorylation by SIAH2-mediated elimination of TYK2 should decrease expression of its target genes, e.g., of *BCL-XL* and *MCL1* [31]. Consistent with the repression of STAT3 phosphorylation (Fig. 5A), expression of BCL2 family proteins decreased significantly upon p53 induction in H1299 cells (Fig. 5B). The functionality of p53 in these cells could be verified as accumulation of the p53 target gene p21^WAF1/CIP^.

Catalytically active TYK2 is necessary for full activation of STAT1-4 in hematopoietic cells treated with type I IFNs [32]. Moreover, TYK2 is essential for the surface expression of IFNAR [10, 33]. We therefore asked if p53 can also affect IFN-induced phosphorylation of STAT1 and STAT3 in lung cancer cells. Indeed, IFNα evoked phosphorylation of both STATs to a significantly lesser extent in H1299 cells expressing p53 (Fig. 5C). Immunoblots for STAT1, STAT3, and several loading controls disfavor a general protein loss or broad transcriptional repression in such cells. These results are consistent with the inverse relationship between SIAH2 levels and STAT-dependent gene expression (Fig. 3).

### Expression of SIAH2 in primary lung tumors

Next, we asked if SIAH2 expression can be correlated with lung carcinogenesis. We constructed tissue microarrays to explore SIAH2 expression in primary lung tumors and normal lung tissue. To increase the reliability of these analyses, we assessed patient samples from three independent cohorts (Jena, Leipzig, and Vienna).

**Fig 6:**
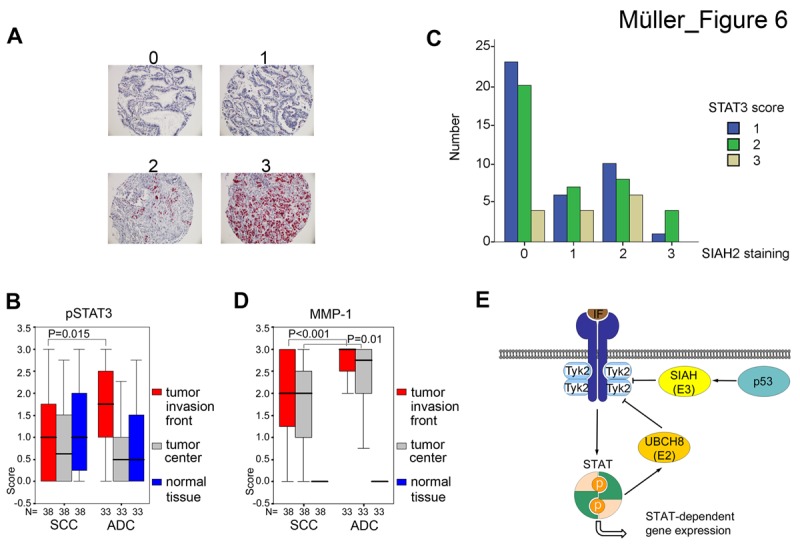
SIAH2 is a marker for SCC and correlates with pSTAT3-dependent MMP-1 expression (A) Examples of SIAH2 protein expression in lung tumor tissues. Scores are defined as 0, SIAH2 negative; 1, weakly positive; 2, moderately positive; 3, strongly SIAH2 positive; magnification 200×. See Table 1 for details on the study cohort from Jena. (B) Comparison of pSTAT3 expression in SCC and ADC (cohort from Leipzig). Significant differences were found for SCC *versus* ADC (Mann-Whitney-U-Test): pSTAT3 is higher in the invasive front (P=0,015). Boxplots show higher pSTAT3/MMP-1 expression in ADC (see also 6D). (C) Analyzing 161 ADC patients (Vienna patient cohort), we found that SIAH and pSTAT3 immunostainig showed an inverse correlation (Chi Square test, p<0,05). Y-axis: number of pSTAT3 positive cases (1 lowest to 3 highest). X-axis: SIAH2 staining intensity (0 lowest to 3 highest). (D) Same as in B, except that MMP-1 was detected. (B/C) Significant differences were found for SCC *versus* ADC (Mann-Whitney-U-Test): MMP-1 is higher in the invasive zone (P<0,001) and tumor center (p = 0,010). Boxplots show higher pSTAT3/MMP-1 expression in ADC (see also 6B). (E) Schematic model summarizing the functions of UBCH8, SIAH2, and p53 for the regulation of TYK2's turnover. IFN-dependent STAT activation and mechanisms regulating expression levels of the E2 ubiquitin conjugase UBCH8 and E3 ubiquitin ligases are indicated. Arrows designate activation and flat-end lines designate repression.

We noted that 71 out of 99 cases (71.7%) were positive for SIAH2 with cytoplasmic staining, while 28 further specimens (28.3%) showed no detectable expression (Fig. 6A and Table 1). A difference of SIAH2 expression between SCC and ADC reached statistical significance with SCC being more frequently positive for SIAH2 compared to ADC (35/40 SCC and 29/52 ADC, P=0.001; Table 1; cohort from Jena, Germany). Similar results were found when 161 further, independent NSCLC cases were analyzed (data not shown; cohort from Vienna, Austria).

**Table 1:** Study cohort for the analysis of SIAH2 expression in NSCLC samples 64 tumor samples were positive for SIAH2, while 28 other cases exhibited no expression. A difference between squamous cell carcinomas (SCC) and adenocarcinomas (ADC) reached statistical significance, with SCC being more frequently positive for SIAH2 (P=0.001).

Detection of SIAH2	negative	positive	p-value
	n (%)	n (%)	
Total No.	28 (28.3 %)	64 (71.7 %)	
SCC	5 (5.4 %)	35 (38 %)	0.001
ADC	23 (25 %)	29 (31.5 %)	

These data and our results showing that SIAH2 accelerates proteasomal degradation of TYK2 and signaling to STAT3 (Fig. 3), prompted us to compare pSTAT3 in ADC and SCC. The presence of tyrosine phosphorylated STAT3 is taken as a surrogate marker as anti-TYK2 antibodies we tested did not work reliably in IHC (data not shown). This analysis revealed that ADC showed more pronounced pSTAT3 staining and this was most pronounced at the tumor invasion front (P=0.015; 38 SCC, 33 ADC; Fig. 6B; cohort from Leipzig, Germany).

Furthermore, analyzing 161 further ADC patients (cohort from Vienna; ADC were chosen as the SCC cases had no pSTAT3 signals sufficient for analyses), we could corroborate an inverse relationship between the expression of SIAH2 and the phosphorylation of STAT3. 53 (50%) cases stained positive for SIAH2, 46 (43%) cases showed immunoreactivity for pSTAT3. SIAH2 and pSTAT3 immunostaining showed an inverse, statistically significant correlation (p<0,05) (Figure 6C).

Matrix metalloproteinase 1 (MMP-1) is associated with lung cancer growth and invasiveness [34], and STAT3 aberrantly induces the *MMP-1* gene in colorectal carcinomas [35]. An assay with the *MMP1* promoter placed in front of luciferase revealed that increasing amounts of SIAH2 repressed the activity of this reporter (Fig. S4). Comparison of MMP-1 in the 38 SCC and 33 ADC samples, accordingly demonstrated that higher pSTAT3 levels in ADC tie in with higher protein levels of MMP-1 *in vivo* (P<0.001) (Fig. 6D).

In summary, these data suggest that higher SIAH2 levels correspond to lower pSTAT3 and less MMP1 expression in human lung cancers. Fig. 6E summarizes key novel findings we present here.

## DISCUSSION

Lung cancer affects ~2 million people worldwide. It is the most common cause of death from cancer accounting for ~1.3 million deaths annually and a 5-year overall survival rate below 15% [36]. NSCLC of SCC and ADC subtypes comprise ~70-80% of all lung tumors. The discrimination between these subtypes has high clinical relevance for decisions on treatment strategies [13, 14, 36, 37]. For example, therapies with the anti-metabolite pemetrexed or the anti-angiogenic antibody bevacizumab should only be applied to SCC cases due to severe toxicity [37]. The transcription factor TTF1 is a marker for ADC occurring within the peripheral airways and the transcription factor p63 is regarded as a stem cell marker for SCC in the bronchial epithelium [37].

Our work contributes to the classification of NSCLC types and discloses putative candidate targets. We show that high SIAH2 expression is associated with low levels of pSTAT3 in SCC, for which no targeted therapies are available. ADC is nowadays the most prevalent lung cancer type [36]. We reveal that phosphorylated STAT3 is more characteristic for ADC than for SCC. This knowledge may become relevant for future therapies and personalized treatment strategies. We show here that among a limited number of known markers SIAH2 appears to be characteristic for the SCC type within the NSCLC group.

A possible molecular explanation for the higher expression of SIAH2 in SCC relies on the *Homo sapiens SIAH2* gene being located on chromosome 3q25. A screen for chromosomal imbalances between ADC and SCC using comparative genomic hybridization showed that specifically SCC was characterized by an amplification of chromosomes 3q and 12p. This could explain the high SIAH2 levels in these lung tumors [36, 38]. Interestingly, in breast cancer tumors, the expression of SIAH2 increases with tumor grading and this increase in SIAH2 expression is associated with gene copy numbers [28], again hitting the genetic locus where *SIAH2* is located. Additional experiments are required to see whether SIAH expression profiles are a cause or a consequence of e.g., genomic instability, environmentally caused aberrations, and if they contribute to invasive processes.

We reveal that SIAH2 regulates STAT1/STAT3 signaling and proteasomal degradation of TYK2. Inspection of the TYK2 protein sequence discloses several VxP SIAH consensus binding motifs. Such motifs frequently allow SIAH-dependent proteasomal degradation [22, 39, 40]. Further experiments are necessary to clarify how SIAHs recognize TYK2. It is possible that similar to the FLT3-ITD kinase [22], proteasomal degradation of TYK2 is enhanced by tyrosine phosphorylation. Solving this question proves difficult as there is an overlay between enhanced expression of UBCH8 and phosphorylation of TYK2 (Fig. 6E). A recent study found that the kinase activity of TYK2 affected its stability in a mouse model and that catalytically inactive TYK2^K923E^ underwent a more rapid lysosomal degradation pathway [32]. It will be interesting to see whether proteasomal and lysosomal degradation pathways interact or if these two pathways act independently on TYK2. Our finding that STAT1 and STAT3 are not degraded upon induction of UBCH8 or SIAHs is consistent with other E3 ubiquitin ligases catalyzing STAT1 turnover [41].

Our study additionally illustrates that the p53-dependent induction of SIAH2 and the proteasomal degradation of TYK2 are distinct from the elimination of JAKs by SOCS-containing E3 ubiquitin ligase complexes [7, 8, 10]. SOCS proteins are induced by activated STAT molecules [6] and we show that SIAH2 is a target of p53. It is currently unclear whether these E3 ubiquitin ligases may have an impact on each other, if UBCH8 is involved in such processes, and how this might affect their targets.

The SIAH2-dependent proteasomal degradation of TYK2 we report here might represent a possibility to reduce aberrant STAT3 signaling and tumor progression. This could possibly be achieved by the activation of SIAH2 by chemotherapies triggering p53 and by IFNs or deacetylase inhibitors activating the expression of the SIAH2-associated UBCH8 [5, 42]. Recent data demonstrate that inactivating the catalytic activity of TYK2 with novel small molecules can attenuate autoimmunity [43]. However, elimination of TYK2 may yield additional benefits. For example, catalytically impaired JAKs can exert scaffold functions. The autoimmunity-associated TYK2 variants TYK2^I684S^ and TYK2^P1104A^ can rescue signaling defects via their association with other intact JAKs [44]. This also seems to hold true for myeloproliferative neoplasm (MPN). MPN cells can survive despite chronic inhibition of JAK2 through heterodimerization with JAK1 or TYK2 and activation *in trans*. RNA interference revealed that such cells remained dependent on JAK2 protein expression [45]. Thus, it might be necessary to eliminate JAKs to delete their disease-associated effects. T cell acute lymphoblastic leukemia (T-ALL) patients may also benefit from therapies eradicating TYK2 expression. In T-ALL activating TYK2 mutations were recently found to drive a STAT1-dependent activation of BCL2 expression. This mechanism was shown to be essential for leukemic cell survival and the TYK2-STAT1-BCL2 axis was hyperactivated in patients with wild-type TYK2 [46].

SCC might gain a proliferative benefit from high SIAH2 levels because SIAH2 promotes proteasomal degradation of the RAS antagonist SPRY2 [47]. Interestingly, there is some dichotomy between RAS and EGFR mutations in lung tumors [37] and EGFR can promote STAT3 phosphorylation and signaling [15, 48]. The possibility that SCC have lower pSTAT3 levels due to lower epidermal growth factor receptor (EGFR) levels though appears unlikely as SCC are lung tumors that frequently (over)express EGFR [36]. SIAH2 may have distinct impacts on transformation pathways via RAS-SPRY2 or EGFR-STAT3. As we found that transfection of SIAH2 reduces the conversion of the metabolic substrate MTT by cells (data not shown), we conclude that high levels of SIAH2 may equally reduce the vitality of lung cancer cells.

Whether the presence and balance of SIAH2 and pSTAT3 are linked to certain phases of oncogenesis still has to be tested. We reveal that lower SIAH2 levels and higher pSTAT3 in ADC compared to SCC are associated with enhanced MMP-1 expression. MMP-1 increases metastatic spread and EGF shedding to augment EGFR signaling [15, 48]. This process could create a vicious circle for ADC. Consistent with these data, STAT3 significantly contributes to lung tumor progression, invasiveness, and metastasis of ADC [15, 18, 49]. In addition, STAT3 signaling acts in a pro-malignant phase during chronic inflammation propelling tumorigenesis [50]. Furthermore, STAT3 can mediate chemoresistance of lung cancer cells [51] and ADC have a higher metastatic potential than SCC, with three out of four ADC subtypes being invasive [36, 37]. Our data suggest that these variances could be mechanistically linked to SIAH2 levels. The fact that increased levels of MMP-1 in SCC compared to normal tissue were detectable might stem from aberrant activation of the transcription factor AP-1. This complex of JUN and FOS controls MMP-1 expression in concerted action with STAT3 [35, 48].

Because STAT1 belongs to a genetic signature predicting lung cancer patient survival [19], effects of SIAH2 on STAT1 and the immunological control of tumorigenesis are possible, i.e. SIAH2 may act as tumor promoter via blocking STAT1. Such a mechanism may not be restricted to lung cancers. For example, SIAH2 expression correlates with breast tumor progression and malignancy [28] and STAT1 exerts growth-inhibitory properties preventing mammary carcinogenesis in mice [52]. Moreover, recent work documented that activation of STAT1 and STAT3 correlated with a better prognosis for colon cancer patients [53]. It is tempting to speculate that an increased expression of SIAH2 during malignant progression may inactivate tumor barriers. An increased expression of SIAH2 in the progression of lung cell transformation has been documented [47] and this may equally promote tumorigenesis through the inactivation of STAT signaling. However, it also has to be noted that larger numbers of lung cancer patient samples have to be analyzed to draw final conclusions. Further studies can be anticipated on this subject, with the hope to define essential steps of lung carcinogenesis and possibilities for targeted intervention strategies.

## MATERIALS AND METHODS

### Cell Lines, transfections, drugs and chemicals

This information can be found in refs. [22, 23, 25, 54]. Cells were incubated with 10^3^ U IFNα, 2.5-25 ng/ml doxycycline, or 2-10 µM MG132 for time periods indicated in Figure legends.

### Preparation of cell lysates, immunoprecipitation/-blotting, and Luciferase reporter assays

These techniques have been described recently [22, 25]. Inputs are 5-10% of lysates used.

### MTT assay and FACS analyses

Details on these methods can be found in ref. [23, 54].

### Antibodies

Antibodies were from Santa Cruz Biotechnology (HA, sc-7392/sc-805; p21, sc-6246; p53, sc-263; MCL1, sc-819; SIAH2, sc-5507/sc-81787; STAT1, sc-346/sc-417; pSTAT1, sc-7988-R; STAT3, sc-7179/sc-282; pSTAT3, sc-8059; TYK2, sc-5271); Sigma (β-Actin, A2066; FLAG, F3165; α-Tubulin, T5168; Ubiquitin, U5379); Calbiochem (MMP-1, IM35T), Cell Signaling (pSTAT3, 9131; TYK2, 9312); Pharmingen (BclX, 516646); Covance (HA, 11-MMS-101P); Invitrogen (V5, 46-0705) and Abgent (UBCH8, AP2118b).

### Immunostaining for phosphorylated STAT3

This was performed as described by [55] employing a rabbit anti-human polyclonal antibody (phosphotyrosine-Stat3 705; Cell Signaling Technology, Beverly, MA); rabbit immunoglobulins (Vector, Burlingame, CA) were used as a negative control. Immunohistological detection of MMP-1 expression was done as described previously [56]. Briefly, formalin-fixed, paraffin embedded sections (4 µm) were deparaffinized and subsequently treated with 0.3% H_2_O_2_ for 30 min to reduce endogenous peroxidase activity. Sections were incubated at 4°C overnight with a 1:50 dilution of anti-MMP-1 (DakoDiagnostik, Hamburg, Germany) in TBS buffer containing 20% goat serum to block unspecific binding sites, followed by incubation with goat-anti-mouse biotin und streptavidin-horseradish peroxidase (BioGenex, San Ramon, CA) and development with diaminobenzidine. Finally, all specimens were coverslipped using Aquatex (Merck, Darmstadt, Germany). Negative controls were performed using an irrelevant monoclonal antibody.

### Tumor samples, tissue microarray, immunohistochemistry for SIAH2, STAT3 and MMP-1

Figure 6A/Table 1: In total, 99 tumor specimens (52 ADC, 40 SCC, 4 small cell carcinoma (SCLC), and 3 large cell carcinoma of lung (LCLC) were included for this study. All patients underwent surgical operation of lung cancer at the Department of Surgery of Charité University Hospital Berlin from 1995 to 2000. No adjuvant radiotherapy or chemotherapy was performed before surgery.

Figure 6B-C: 108 lung carcinoma biopsies (53 SCC, 43 ADC, 6 LCLC, 3 SCLC, 3 adenosquamous carcinoma) were obtained in the course of tumor resections. All patients underwent surgical operation of lung carcinoma at the University Hospital Leipzig from 1999 to 2004. Due to small sample sizes of SCLC and LCLC these were not further evaluated. All studies were approved by the local ethical committees.

Immunostaining for pSTAT3 is described above. Immunohistochemical staining of the slides was read and scored by the pathologist A. Schütz. A composite three step score system based on the percentage and signal intensity of pSTAT3 in cell nuclei, was used for pSTAT3.

Tissue microarrays (TMAs) and immunohistochemistry for SIAH2 were done as reported [57]. Suitable areas for tissue retrieval were marked on standard haematoxylin and eosin (H&E) sections. Two tissue cylinders per tumor with a diameter of 0.6 mm were present in one TMA. After construction, 4 µm sections were cut from donor blocks and transferred to glass slides without any sectioning aids. Monoclonal mouse anti-SIAH2 antibody was used at a 1:200 dilution. Arrays were read manually by the pathologist Prof. Dr. I. Petersen. Immunohistochemistry was scored semi-quantitatively as negative (score 0), weak (score 1), moderate (score 2), or strong (score 3) as previously described [57]. Weak staining corresponded to faint signal intensities of the tumor cells that were hardly distinguishable from background or unspecific staining. For statistical evaluation, scores 0-1 were therefore considered as negative and scores 2-3 as positive.

Figure 6D: Immunohistochemistry was performed on 4 µm-paraffin sections from one representative tissue block per patient stored at the archives of the Department of Pathology, Medical University of Vienna, Austria, using antibodies against SIAH2, pSTAT3, and STAT3. Negative controls were treated with isotype IgG control antibodies. Immunoreactive tumor cells were counted by K.S. The scoring system integrated intensity and extent of immunostaining: the number of positive tumor cells was scored 0 (no staining), 1 (<10%), 2 (10-50%), 3 (51-80%), and 4 (81-100%). Intensity of staining was scored 0 (negative), 1 (weak), 2 (moderate), 3 (strong). The results of extent and intensity of tumor cell staining were multiplied to assess the final score.

### Statistical analysis

The association between SIAH2 expression and subtypes of NSCLC (SCC and ADC) was analyzed by Fisher's exact test. Wilcoxon rank sum test (Mann–Whitney U test or Mann–Whitney–Wilcoxon Wilcoxon rank-sum test) was applied to determine significant associations between dichotomous variables and pSTAT3 staining. Kruskal-Wallis test was used for variables with more than two categories. Statistical analyses were performed with SPSS 13.0 for windows (SPSS, Inc., Chicago, USA). Difference at P<0.05 was considered statistically significant.

## SUPPLEMENTARY INFORMATION AND FIGURES


