# Supplementary material for: SIAH2 antagonizes TYK2-STAT3 signaling in lung carcinoma cells
Source: Oncotarget. 2014 Apr 12;5(10):3184–96. doi: 10.18632/oncotarget.1899 (PMC4102802; doi:10.18632/oncotarget.1899)
Supplement: Supplementary file 1 [file oncotarget-05-3184-s001.pdf]

SIAH2 antagonizes TYK2-STAT3 signaling in lung carcinoma cells

Supplementary Information

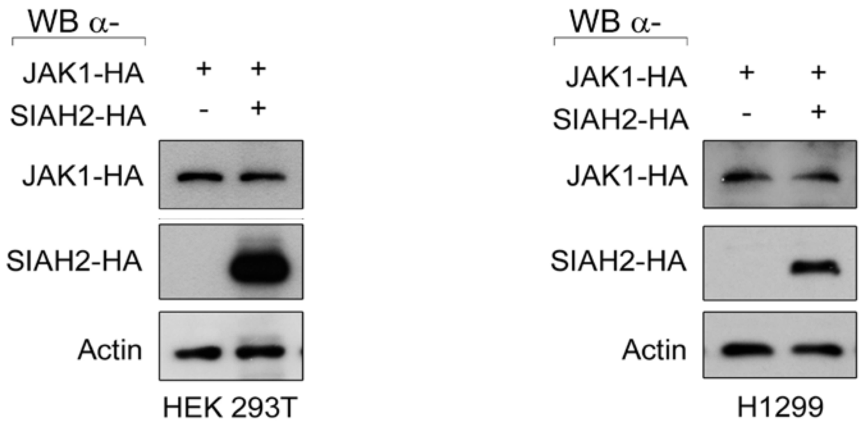

Supp fiure (S1): HEK293T cells (left) and H1299 cells (right) were transfected with vectors encoding HA-JAK1 (1 µg), wild-type SIAH2 (0.5 µg) or empty vector pcDNA3.1 (0.5 µg) instead of SIAH2. Cell lysates were analyzed by Western blot (WB; α, anti) as indicated.

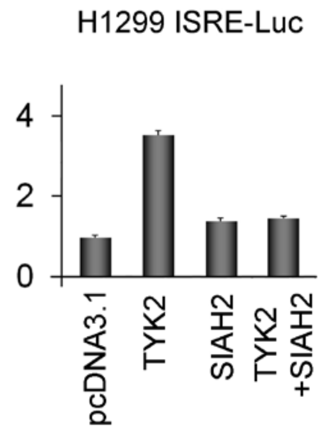

Supp figure (S2): Luciferase assay with an ISRE-Luc construct quantifies transcriptional activity of STAT1/STAT2 heterodimers in H1299 cells. Luciferase activity was normalized to β-Gal activity for each of the triplicate.

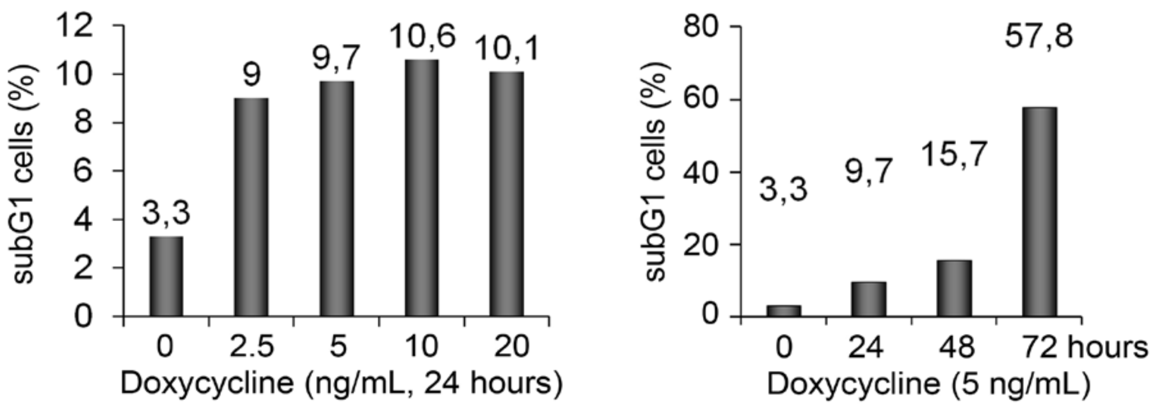

Supp figure (S3): Left: H1299 cells were treated with doxycycline (0-20 ng/ml) or left untreated for 48 h. Then, the cells were trypsinized (Accutase, PAA), washed twice with 1×PBS and stained with propidium iodide. The percentages of apoptotic cells (SubG1) were determined by flow cytometry. Right: H1299 cells were treated with doxycycline (5 ng/ml) or left untreated for 48 h. Then, the cells were trypsinized (Accutase, PAA), washed twice with 1×PBS and stained with propidium iodide. The percentages of apoptotic cells (SubG1) were determined by flow cytometry (numbers state average % of dead cells, N=2).

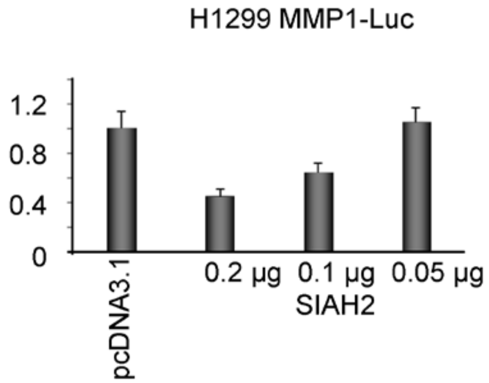

Supp figure (S4): Luciferase assay with an MMP1-Luc construct. H1299 cells were transfected with 0.4 µg MMP-1-LUC, 0.2 µg SV40- β-Gal, 0.05-2 µg SIAH2, or empty vector pcDNA3.1 (empty vector pcDNA3.1 was sued to obtain equal amounts of DNA for transfection). Cells were transfected for 48 h. Luciferase activity was normalized to β-Gal activity for each of the triplicate.
